# Infective Endocarditis with Gerbode Defect and DRESS Syndrome: A Rare Case Report

**DOI:** 10.3390/reports8030127

**Published:** 2025-07-31

**Authors:** Corina Ureche, Diana Lavinia Moldovan, Ionel Vița, Valeria Guila, Teodora Nicola-Varo

**Affiliations:** 1 Second Internal Medical Department, George Emil Palade University of Medicine, Pharmacy, Science and Technology, 540142 Târgu Mureș, Romania; corina.ureche@umfst.ro (C.U.); teodora.nicola-varo@umfst.ro (T.N.-V.); 2 Clinical Emergency County Hospital, 540136 Târgu Mureș, Romania; ciobanu.valeria.23@stud.umfst.ro; 3 Institute of Emergency for Cardiovascular Diseases and Transplant, 540136 Târgu Mureș, Romania

**Keywords:** Gerbode’s defect, DRESS syndrome, infective endocarditis, oxacillin therapy, *Staphylococcus aureus*

## Abstract

**Background and Clinical Significance:** Infective endocarditis (IE) is a serious condition with rising incidence, frequently caused by *Staphylococcus aureus*. However, cases involving rare congenital anomalies such as Gerbode’s defect are uncommon. **Case Presentation:** This report presents the first documented case of IE in a patient with a congenital Gerbode defect complicated by DRESS syndrome—a severe, drug-induced hypersensitivity reaction typically triggered by antibiotics like oxacillin. A 65-year-old woman developed infective endocarditis involving vegetations on the cardiac device lead, the tricuspid valve, and adjacent to a Gerbode defect. The diagnosis was confirmed by positive blood cultures and echocardiographic findings. She received treatment with oxacillin. Subsequently, she exhibited clinical features consistent with DRESS syndrome, including rash, eosinophilia, and multi-organ involvement. Rapid recognition and management, including corticosteroid therapy and antibiotic modification, led to clinical improvement. **Conclusions:** This case highlights the importance of vigilance for DRESS syndrome in prolonged antibiotic therapy for IE, especially in the context of rare congenital cardiac anomalies. In addition, guidelines are needed to optimize the diagnosis and treatment of this potentially lethal complication.

## 1. Introduction and Clinical Significance

Infective endocarditis (IE) is a life-threatening condition with severe complications. The incidence of IE has increased over the last 30 years [1,2]. Although *Staphylococcus aureus* is the most common cause [3,4], it has very rarely been reported to be complicated by DRESS syndrome (Drug Reaction with Eosinophilia and Systemic Symptoms) during oxacillin therapy in the setting of treatment for infective endocarditis [5,6,7].

DRESS syndrome is a potentially life-threatening, drug-induced hypersensitivity response, established by a severe idiosyncratic reaction with eosinophilia, fever, diffuse rash, lymphadenopathy, and plurivisceral involvement. The drug-immune reaction is T-cell-mediated [8] and usually occurs within 2 to 6 weeks following the administration of the drug [7,8,9,10]. While the most common drugs involved in DRESS syndrome are anticonvulsants and allopurinol, oxacillin-induced cases have been reported very rarely [11,12].

While valvular or cardiac device involvement in IE is more common, we reported a case with an extremely rare involvement of infective endocarditis—the Gerbode’s congenital defect [13,14]. The Gerbode’s defect is a rare ventricular septal defect creating a communication between the left ventricle and right atrium [15,16]. The congenital Gerbode defect is an extremely rare (0.08%) type of ventricular septal defect and represents less than 1% of all congenital cardiac defects [17,18]. To the best of our knowledge, this is the first reported case of infective endocarditis involving a congenital Gerbode defect complicated by DRESS syndrome.

Therefore, given the long period of antibiotic therapy required for the treatment of infective endocarditis [2], the recognition and management of DRESS syndrome, a potentially lethal condition, are essential, even if sometimes it mimics sepsis with leukopenia [19].

## 2. Case Presentation

### 2.1. Patient Information

#### 2.1.1. Demographics

The individual in this case report was a 65-year-old Romanian female and a lifelong resident of Romania.

#### 2.1.2. Presenting Concerns

The patient was admitted to the Psychiatric Department following a ground-level fall. Known as having a psychiatric diagnosis, the event of the fall was accompanied by complex visual and auditory hallucinations, paranoid delusional ideation, significant temporal and spatial disorientation, marked bradyphrenia, confusional episodes, anxiety, major depressive episodes, and psychomotor agitation. During hospitalization, she exhibited a decline in general condition, psychomotor slowing, fever, diaphoresis, fatigue, arterial hypotension, and tachycardia and she has been transferred to the Institute of Emergency for Cardiovascular Diseases and Transplant.

### 2.2. Medical History

The patient’s medical journey began with significant interventions, notably the implantation of a permanent single-chamber pacemaker in 1985 to address a congenital complete atrioventricular block. Due to battery depletion, pacemaker generator replacements were carried out in 1995, 2004, and 2012. A right ventricular pacing lead fracture in 2016 necessitated the reimplantation of a new dual-chamber pacing system, preserving the original ventricular lead. Persistent atrial fibrillation was effectively converted to sinus rhythm through external electrical cardioversion.

In addition to these interventions, the patient’s medical history includes several chronic conditions: a minor interventricular septal defect, mild mitral and tricuspid valve regurgitation, arterial hypertension, hypercholesterolemia, paranoid personality disorder, mixed anxiety-depressive disorder, mild cognitive impairment, multinodular goiter, osteoporosis, and chronic constipation. The patient has a history of chronic tobacco use but is now a former smoker.

Family and Psychosocial History: No significant family and psychosocial history has been noted.

Prior Medication: Regarding prior medication usage, the patient has been on a regimen that includes Rivaroxaban 20 mg once daily, Amiodarone 100 mg once daily, Ramipril 10 mg once daily, Tianeptine Sodium 12.5 mg twice daily, Alprazolam 0.25 mg three times daily, and Cebrium once daily.

### 2.3. Clinical Case

**Figure 1:**
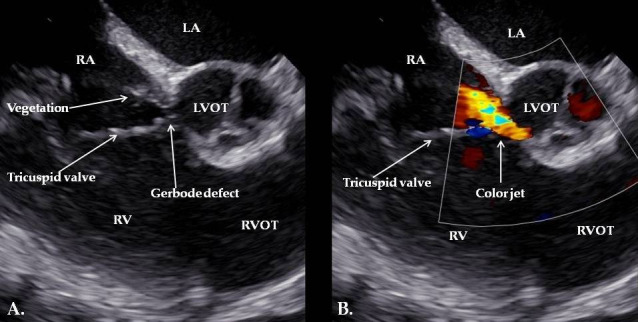
Two-dimensional transesophageal echocardiogram shows a Gerbode defect, along with a mobile, echogenic and filiform mass attached near the tricuspid valve (measuring 19 × 6 mm) (**A**). Color Doppler imaging demonstrates an abnormal, high-velocity systolic jet extending from the left ventricle into the right atrium. This finding confirms the presence of a left ventricle-to-right atrium communication, characteristic of a Gerbode defect (**B**). LA: Left Atrium; RA: Right Atrium; RV: Right Ventricle; LVOT: Left Ventricular Outflow Tract; RVOT: Right Ventricular Outflow Tract.

**Figure 2:**
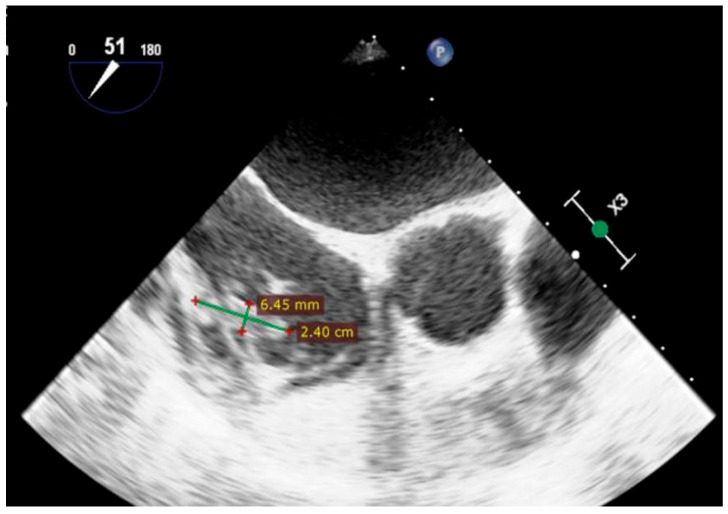
Two-dimensional transesophageal echocardiogram shows a large vegetation attached to one of the pacemaker leads, measuring 24 mm × 6.45 mm. The vegetation is measured and indicated with green crosses.

A 65-year-old woman was admitted through the emergency care room to the Psychiatric Department due to a psychiatric condition, which included paranoia, anxiety, cognitive decline, and major depressive episodes. During hospitalization, she developed signs of infection, including hypotension (75/40 mmHg), fever (40 °C), and leukocytosis (12,400/µL leukocytes, 11,000/µL neutrophils, respectively 91%, eosinophils 0.02%). Due to her medical history, she was transferred to the Cardiology Institute, and the causes of the infection were investigated. Blood cultures were taken, CT scans showed post-tuberculosis lung scarring, urine examination, and sequential sputum samples from bronchoscopy were negative, including tuberculosis (Tabel 1). A transesophageal echocardiography was performed, which revealed a congenital perimembranous ventricular septal defect (Gerbode’s defect) with a left-to-right shunt, a mobile vegetation attached, close to the tricuspid valve (measuring 19 × 6 mm) (Figure 1), a second vegetation involving the tricuspid valve (measuring 11 × 12 mm), and a third vegetation on the pacemaker lead (measuring 24 × 6.45 mm). Therefore, the cause of the infection was identified as an infective endocarditis (IE) (Figure 2).

**Table 1:** Timeline of events and interventions.

Day	Stage	Clinical Manifestations	Diagnostic Workup	Therapeutic Interventions
2 weeks before	Initial Presentation	Paranoia, anxiety, cognitive decline, major depressive episode		Risperidone 2 mg od, Escitalopram 10 mg od
Days 1–2	Infection Onset	Hypotension, fever, leukocytosis	Blood cultures—*Staphylococcus aureus* TTE—suspicion of endocarditis due to mobile hyperechoic masses identified CT scans of the chest, abdomen, and pelvis showing post-tuberculosis lung scarring	
Day 3	Diagnosis of IE	Persistent fever, systemic inflammation	TEE: Gerbode defect with a vegetation attached near the tricuspid valve and others involving the tricuspid valve and the pacemaker lead	
Days 4–27	Antibiotic Therapy			Gentamicin 3 mg/kg/day + Oxacillin 12 g/day (dual therapy for 2 weeks), then Oxacillin monotherapy for 2 more weeks);
Day 27	Development of DRESS Syndrome	Rash, fever, leukopenia, eosinophilia, organ involvement	RegiSCAR score assessment; procalcitonin level	discontinuation of Risperidone; antibiotic switch to Linezolid + Ceftazidime
Day 28	Further Investigations	Persistent symptoms, eosinophilia	Exclusion of fungal superinfection, hematologic disease, serum sickness, repeat blood cultures	
Day 30	Corticosteroid Therapy	Skin rash and eosinophilia		Dexamethasone 8 mg/day for 4 days
Day 31+	Pacemaker-related complications	Vegetation on pacemaker lead	TEE: Persistent vegetations confirmed; lead extraction via transvenous approach	Temporary external pacemaker; continued antibiotic therapy
Week 6	Recovery and Stability	Overall clinical and laboratory improvement	Echocardiography: ejection fraction; coronary angiography	Evaluation for potential cardiac resynchronization therapy
Day 38	Blood Disorders	Anemia and thrombocytopenia	Upper and lower GI endoscopy; platelet function tests; vitamin B12/folate levels	-
Week 9	Spontaneous Remission	Resolution of hematologic abnormalities,	-	
6-month Follow-up	Long-term Outcome	asymptomatic, no signs of recurrence or disease progression	The cardiology follow-up visit; clinical and biochemical stability	Routine follow-up

Methicillin-sensitive *Staphylococcus aureus* was isolated from blood cultures and found to be sensitive to oxacillin and gentamicin. Extended therapy was initiated for the treatment of IE. The treatment plan included gentamicin at 3 mg/kg/day IV in two doses and oxacillin at 12 g/day IV in six doses, administered simultaneously for the first two weeks, followed by oxacillin alone for an additional two weeks.

**Figure 3:**
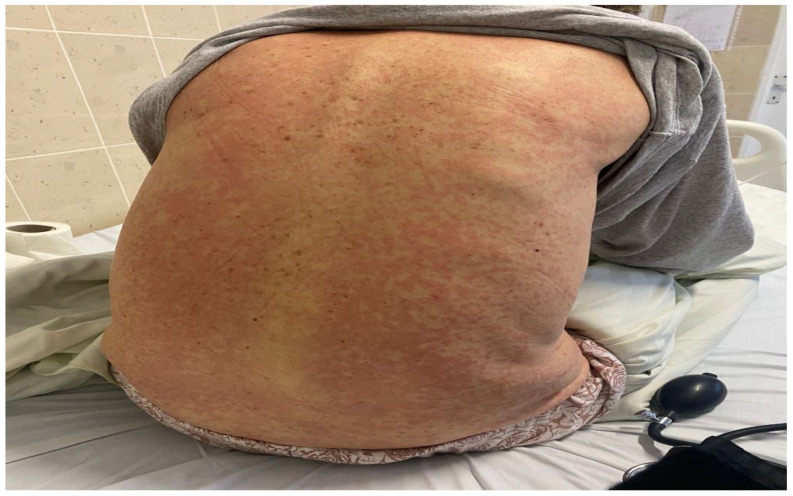
An extensive erythematous rash involving the posterior trunk, consisting of diffuse macules and papules, coalescing into larger patches in some areas.

**Figure 4:**
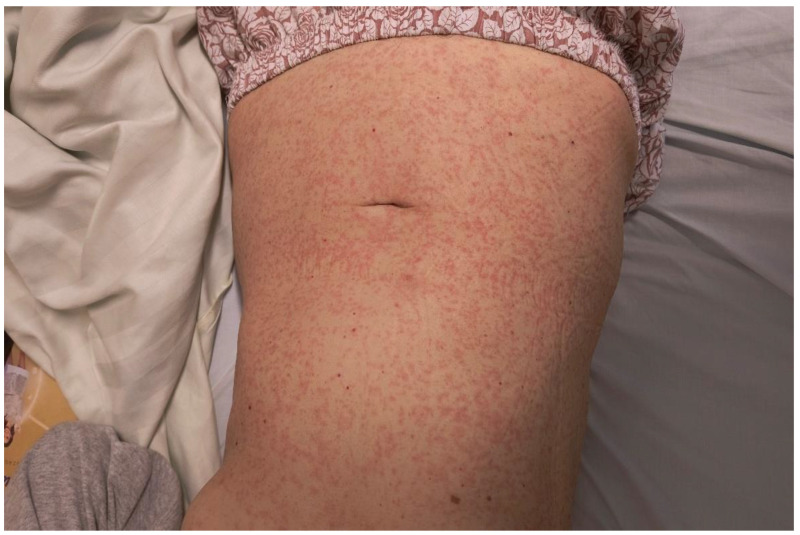
Diffuse skin rash with significant distribution over the chest and abdomen. Despite marked inflammation, the epidermis remains intact, with no evidence of blistering or necrotic changes.

On the 27th day of therapy, the patient developed an extensive cutaneous rash involving the chest, trunk, abdomen, upper and lower extremities, with periorbital edema and fever (39 °C) (Figure 3 and Figure 4). Laboratory results revealed leukopenia (850/µL), severe neutropenia (190/µL), eosinophilia (340/µL, respectively 33%), increased serum transaminases (AST 95 U/L, ALT 41 U/L), and creatinine (1.7 mg/dL).

The European Registry on severe cutaneous adverse drug reactions (RegiSCAR) was used to assess the likelihood of DRESS syndrome as the primary diagnosis and received a score of 6 points (fever of 39 °C, eosinophilia of 340/µL-33%, skin rash extent > 50%, edema, infiltration, affected kidney, liver, as well as hematologic abnormalities: leukopenia, anemia, severe neutropenia, thrombocytopenia, alternative diagnoses excluded with three biological investigations, resolution delay greater than 15 days).

We took into consideration the possibility of sepsis and performed a procalcitonin test, which returned a value of 0.79 ng/mL (normal). The possibility of a febrile neutropenia was also taken into consideration. To complete the 6-week course of antibiotics, the treatment was switched from oxacillin to linezolid and ceftazidime, according to the antibiogram. We also considered a fungal superinfection, which returned negative results. A blood smear has been performed to exclude a hematologic disease. We ruled out the possibility of staphylococcal granulomas or other pulmonary lesions based on imaging. The psychiatric medication, risperidone, that could potentially cause leukopenia has been discontinued and ruled out as the cause, since after reinitiating it a few days later, there were no clinical or laboratory changes. Also, we considered the differential diagnosis of serum sickness, given the presence of rash and fever, and we tested for circulating immune complexes with a positive result (113 UF). Taking into account the absence of polyarthritis or polyarthralgia, the presence of eosinophilia, the involvement of multiple organs, the RegiSCAR score of 6 points, time of symptoms appearance correlated with drug administration, and the possibility of elevated circulating immune complexes in the context of endocarditis [20], the serum sickness diagnosis was abandoned.

On the 28th day, following the previously mentioned steps, a slight improvement in the white blood cell count was noted (2240 leukocytes/µL, 690 neutrophils/µL). By day 30, the leukocyte levels normalized, but eosinophilia worsened (from 340/uL-33% to 1020/uL-45.5%) and the cutaneous rash persisted. At this stage, to enhance recovery from DRESS syndrome, a short course of systemic corticosteroids was started, involving Dexamethasone at a dose of 8 mg per day for four days. Thereafter, the skin rash also showed significant signs of improvement, with gradual remission observed in laboratory investigation.

Due to the presence of vegetation on one of the leads in the atrial portion, a transvenous extraction of the dual-chamber pacemaker and a passive fixation ventricular pacing lead was performed. Due to congenital complete atrioventricular block and pacing dependency, a pacing lead was placed in the right ventricle and connected to a temporary single-chamber external pacemaker attached to the skin. Antibiotic therapy (guided by the results of the antibiogram, which indicated sensitivity to these agents) was continued for the next three weeks with linezolid, ceftazidime, and fluconazole IV. In the following days, a pacing malfunction occurred (intermittent capture, hemodynamic instability) due to lead displacement from the right ventricle, requiring reintervention for lead repositioning.

Three weeks after changing the antibiotics, the patient’s cutaneous state improved and the white blood cell count began to decrease again (remaining within the lower normal limits). Given the patient’s stable clinical and laboratory status—no recurrent fever, negative inflammatory markers, and consecutive negative blood cultures—antibiotic therapy was discontinued. On the 38th day, the patient presented with anemia and thrombocytopenia, almost stationary with hemoglobin of 7.3 g/dL and 77,000–100,000 platelets/µL despite the administration of erythrocyte mass, without sanguine externalization. Upper and lower gastrointestinal endoscopy were performed, without identifying a source of bleeding. We collected platelets using a citrate-based medium, considering a possible pseudothrombocytopenia due to an EDTA reaction medium, yielding a similar result. Ferritin, serum iron, transferrin, folic acid, and vitamin B12 tests were performed, which revealed normal values except for low folic acid levels. We performed direct and indirect bilirubin tests, LDH and Coombs test to rule out hemolytic anemia, all of which returned normal results. In week number 9, spontaneous remission of anemia (Hgb 11.9 g/dL) and thrombocytopenia was observed, with the previous episode interpreted as a possible complication of DRESS syndrome overlapping with infective endocarditis.

## 3. Follow-Up and Outcomes

A transthoracic echocardiogram performed at follow-up demonstrated a reduced left ventricular ejection fraction of 29%, estimated by the biplane Simpson method, and evidence of contractile dyssynchrony, while coronary angiography revealed no significant coronary artery disease. Given the patient’s dependence on pacing due to complete atrioventricular block, cardiac resynchronization therapy (CRT) is being considered going forward.

At the 6-month follow-up, the patient remained clinically stable and asymptomatic, with no evidence of disease progression or recurrence. Regular follow-up appointments were scheduled to ensure ongoing clinical and biochemical stability.

## 4. Discussion

The Gerbode defect is an uncommon cardiac malformation, named after Frank Gerbode, the pioneering surgeon who first documented a successful series of cases in which patients underwent surgery for a left ventricle-to-right atrium shunt in 1958. It is characterized by a peri membranous ventricular septal defect, which creates an abnormal connection between the left ventricle and the right atrium. This condition can be present in both congenital and acquired forms, with the congenital type being rarer. Acquired cases can develop as a result of trauma, surgical interventions, inferior myocardial infarction, or infective endocarditis [15,21,22]. In contrast, a congenital Gerbode defect complicated by endocarditis—rather than being provoked by it, as in acquired cases—is extremely uncommon, with only a few documented cases [14,23].

Although the patient had a congenital Gerbode defect prior to the onset of infective endocarditis, its distinct anatomical and hemodynamic features likely contributed to increased susceptibility. Vegetations typically develop in areas where pressure gradients create turbulent blood flow. In Gerbode defects, the high-velocity left ventricle-to-right atrium (LV–RA) shunt generates significant turbulence and mechanical stress on the endocardial surface, potentially leading to localized endothelial injury. These structural and flow-related abnormalities provide a favorable environment for bacterial adhesion and infection. Compared to more common congenital heart defects with lower-velocity shunts, the direct LV–RA jet in Gerbode defects is associated with more severe endothelial disruption, thereby increasing the risk of infective endocarditis [14,24,25].

Oxacillin-triggered DRESS syndrome is an exceptionally uncommon condition. We found only a single previously documented case of DRESS syndrome linked to oxacillin administration and associated with agranulocytosis. To our knowledge, this is the first reported case of infective endocarditis complicated by oxacillin-induced DRESS syndrome with leukopenia, as well as the first known case of infective endocarditis affecting a congenital Gerbode defect, which was further complicated by DRESS syndrome [5,6,7,14].

DRESS syndrome is a severe condition with a high mortality rate [26], making early detection and prompt treatment essential to preventing life-threatening consequences. The diagnosis should be suspected in any patient presenting with a cutaneous rash, fever, eosinophilia, edema, and multi-organ involvement within two to six weeks of starting a medication.

The relationship between organ involvement and its management is essential, along with the need to investigate differential diagnoses such as alternative causes of anemia, contrast-induced kidney injury (as observed in our case), liver dysfunction, and hypoalbuminemia, whether due to endocarditis or other comorbidities. Visceral organ dysfunction is a leading cause of morbidity and mortality in these patients. Elevated serum transaminases, hypoalbuminemia, and serum creatinine levels exceeding more than twice the patient’s baseline suggest hepatic and renal impairment, which are the most commonly affected organs [27]. Moreover, anemia associated with thrombocytopenia, after excluding other potential causes, may represent a delayed complication of DRESS syndrome. The RegiSCAR score can be utilized to estimate the probability of DRESS syndrome.

Laboratory investigations, such as a complete blood count with differential and peripheral smear, along with renal and hepatic function tests, are essential for establishing the diagnosis, excluding other potential causes, and assessing the extent of visceral organ involvement. Moreover, serial blood cultures, including fungal cultures, should be performed to detect possible fungal superinfections in endocarditis, particularly in cases where fever persists despite ongoing antibiotic therapy.

The delay between drug initiation and symptom onset presents a major diagnostic challenge. Once the complication occurs, the primary approach in managing DRESS syndrome is to either discontinue the antibiotic or, in our case of infective endocarditis, switch to an alternative antibiotic. Current expert consensus highlights the importance of prompt withdrawal of the causative and cross-reacting drugs, initiation of supportive care including fluid and electrolyte management, hemodynamic balance, supportive therapy, fever management, careful organ monitoring, gastric protection, and avoidance of empirical use of NSAIDs or antibiotics [28,29]. We attempted to manage target organ damage through fluid and electrolyte rebalancing and intravenous amino acid solution infusion until renal and hepatic function normalized. Red blood cell transfusions were administered multiple times, along with serial complete blood counts, to correct severe anemia. Albumin administration was avoided after the diagnosis of DRESS syndrome to prevent potential worsening of the condition. Furthermore, there is a shortage of research providing clear guidelines for managing DRESS syndrome when an infection is ongoing. The management of DRESS syndrome becomes particularly complex when it occurs in the setting of a severe concomitant infection such as infective endocarditis. In such cases, the treatment must balance the need to control the immunoallergic response with the risk of immunosuppression that could exacerbate the underlying infection. Systemic corticosteroids remain the most widely accepted treatment for moderate to severe DRESS syndrome, particularly in the presence of visceral involvement such as renal, hepatic, or pulmonary dysfunction. However, in the context of active infective endocarditis, the use of corticosteroids presents a therapeutic challenge and must be cautiously considered. In the case of our patient, a reduced corticosteroid dose and shorter duration were chosen to mitigate the risk of worsening sepsis, while still aiming to control the inflammatory response associated with DRESS syndrome. This tailored approach is supported by recent literature, which suggests that corticosteroid regimens can be adjusted based on infection severity, provided close clinical and laboratory monitoring is maintained. Additionally, early multidisciplinary involvement and longitudinal follow-up are essential not only to guide acute management, but also to anticipate potential long-term autoimmune sequelae, which may develop months to years after apparent resolution [30,31,32,33].

Additionally, leukopenia is rarely associated with DRESS syndrome; therefore, we consider the immediate recognition of the diagnosis essential. Given the presence of leukopenia, we considered it appropriate to transfer the patient to an isolated room with aplasia-specific precautions. While leukocytosis is more commonly reported, recent evidence—including a large study published in the World Allergy Organization Journal— highlight that leukopenia and neutropenia frequently occur early, affecting over two-thirds of patients. Additionally, case reports have documented leukopenia occurring shortly after DRESS syndrome onset, suggesting it may be an underrecognized early hematologic sign [34].

Special consideration should be given to the potential complications of DRESS syndrome in IE, as the extended antibiotic therapy required for endocarditis may elevate the risk. Furthermore, the clinical and laboratory overlap between DRESS syndrome and infective endocarditis can lead to delayed recognition and treatment.

Moreover, our case required extensive interdisciplinary coordination, involving specialists from cardiology, allergology, arrhythmology, psychiatry, infectious diseases, cardiovascular surgery, interventional cardiology, laboratory medicine, radiology, internal medicine, pulmonology, gastroenterology, and hematology. Just as essential was the dedicated and continuous care provided by the nursing staff throughout the hospitalization.

## 5. Conclusions

This case illustrates the importance of early recognition of DRESS syndrome as a serious complication of prolonged antibiotic therapy in infective endocarditis (IE). The atypical presentation with leukopenia, carrying a risk of progression to agranulocytosis, highlights the need for heightened clinical vigilance when patients develop rash, fever, leukopenia, and eosinophilia.

To our knowledge, this is the first reported case of infective endocarditis in a congenital Gerbode defect complicated by oxacillin-induced DRESS syndrome, emphasizing the rarity and clinical challenges of such a presentation. The complexity and severity of this case highlight the need for well-established diagnostic and therapeutic guidelines for DRESS syndrome, particularly in patients undergoing prolonged targeted antibiotic therapy for infective endocarditis.

## Data Availability

The original contributions presented in this study are included in the article. Further inquiries can be directed at the corresponding authors.

## Abbreviations

The following abbreviations are used in this manuscript:

IE	Infective endocarditis
DRESS	Drug Reaction with Eosinophilia and Systemic Symptoms
RegiSCAR	The European Registry on severe cutaneous adverse drug reactions
TTE	Transthoracic echocardiography
